# Functionality and Acceptance of the EsoCap System—A Novel Film-Based Drug Delivery Technology: Results of an In Vivo Study

**DOI:** 10.3390/pharmaceutics13060828

**Published:** 2021-06-02

**Authors:** Christoph Rosenbaum, Michael Grimm, Julius Krause, Adrian Rump, Rebecca Kessler, Norbert Hosten, Werner Weitschies

**Affiliations:** 1 Department of Biopharmaceutics and Pharmaceutical Technology, Institute of Pharmacy, University of Greifswald, Felix-Hausdorff-Straße 3, 17489 Greifswald, Germany; christoph.rosenbaum@uni-greifswald.de (C.R.); michael.grimm@uni-greifswald.de (M.G.); julius.krause@uni-greifswald.de (J.K.); adrian.rump@uni-greifswald.de (A.R.); 2 Institute of Diagnostic Radiology and Neuroradiology, University Medicine Greifswald, Ferdinand-Sauerbruch-Straße, 17475 Greifswald, Germany; rebecca.kessler@uni-greifswald.de (R.K.); hosten@uni-greifswald.de (N.H.)

**Keywords:** drug delivery, eosinophilic esophagitis, esophagus therapy, esophageal diseases, MRI study, ease of swallowing, user perspective

## Abstract

There are no methods for specific local application of active substances to the mucosa of the esophagus to treat eosinophilic esophagitis or other esophageal diseases. This publication describes the principal in vivo functionality and acceptance of a novel modular drug delivery concept, called EsoCap system, by 12 healthy volunteers. For the first time, the EsoCap system enables targeted placement on the esophageal mucosa of a mucoadhesive polymer film. Acceptance was determined by means of a standardized questionnaire after administration and functionality of the device by MRI scans. Two different setups of the EsoCap system were tested: one setup with a density of 0.4 g/cm^3^ and one with a density of 1.0 g/cm^3^. Acceptability of the dosage form was also confirmed in addition to functionality, by measuring the applied film length. It was found that acceptance of the variant with the higher density was significantly better. This novel drug delivery technology could enable a targeted, local and long-lasting therapy of the esophagus for the first time, depending on the polymer film used.

## 1. Introduction

The esophagus is a muscular tube, about 25 cm long, that transports a swallowed bolus into the stomach with peristaltic movements, by means of striated muscles in the first third and smooth muscles in the two lower thirds. The trachea is thereby closed via the laryngeal inlet to prevent aspiration of food components [1]. The transit time of drugs or food components through the esophagus varies between a few seconds for liquids and up to 120 s for pieces of meat [1,2]. It depends on the bolus size, body posture, individual physiology and the amount of liquid used for intake [1,2]. Transit times of 3 s to 15 s can be expected for solid oral dosage forms [1,2]. These ultra-short transit times are particularly problematic for the treatment of localized diseases. Topical glucocorticoids, such as budesonide or fluticasone, are used for the treatment for eosinophilic esophagitis. Local therapy in the esophagus places particularly high demands on the dosage form. It has been shown by Dellon et al. that maximizing the dosage form mucosal contact time in the esophagus is directly associated with greater clinical efficacy [3]. Previous easily ingestible therapies have proven difficult due to the extremely short transit times described above. Examples of previously used forms of administration are the off-label use of inhalers and use of syrups or suspensions containing active substances, such the “Jorveza” orodispersible tablet [4,5,6,7].

An innovative solution to the problem of short contact times for topically applied dosage forms in the esophagus has been described and developed by Krause et al. [8]. The principle of the EsoCap esophageal drug delivery system is a thin hydrophilic polymer film that absorbs water on contact with moist mucous membranes, becomes sticky and for this reason adheres to the mucosa.

**Figure 1:**
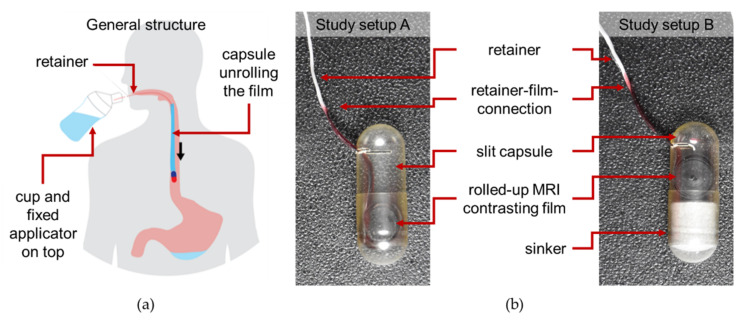
Structure and functionality of the EsoCap system tested. (**a**) General structure of the entire system, with the drinking cup and applicator fixed to it. The retainer attached to the applicator and, at the other end, to the film triggers unrolling of the film from the capsule. (**b**) Study setup A: Slit capsule (size 00) filled with a rolled hibiscus tea contrasting PVA film. Study setup B: A sinker-extended EsoCap system to increase the system’s density was intended to improve swallowability/functionality.

The EsoCap system generally consists of a slit capsule, a thin polymer film, a string, a sinker (to increase the density of the system; setup B only), an applicator, and a drinking cup (Figure 1). The polymer film is rolled up in a conventional hard capsule, which has a slit from which the end of the polymer film protrudes. The basic idea of the novel dosage form is that the polymer film is pulled out of the capsule during the swallowing process, comparable to an adhesive tape dispenser. The EsoCap system film is the adhesive tape that adheres to the esophageal mucosa due to its mucoadhesiveness. The capsule shell, with the sinker contained therein for better swallowability, disintegrates in the stomach [9]. To provide the traction necessary for unrolling and to control unrolling in the esophagus, only the polymer film is attached to a thin string of a defined length, which is subsequently referred to as the retainer. The retainer is connected at its open end to a special 3D printed applicator. This is screwed onto a suitable drinking cup, filled with water before intake. This novel dosage form should thus allow targeted application of active substances, via the film, to the esophageal mucosa.

The main potential advantage of this application could be achievement of high local concentrations of active substances, which should lead to improved efficacy, to a reduced frequency of application and to reduced side effects as well. The application can also increase drug retention time on the esophageal mucosa, which is important for the therapy’s success [3].

Placement of a polymer film in the esophagus is not associated with unpleasant perceptions, as mechanical sensitivity is not very high in the esophagus; the literature even describes retention of large objects in the esophagus without perception by subjects [10].

The application of capsules attached to a string is already established in the field of esophageal diagnostics (Cytosponge™) [11]. Moreover it is well known from various studies how taking oral dosage forms is a daily challenge for many patients [12,13,14,15,16]. In this context, Schiele et al. describe not only the general problem of ingestion, but also the prevalence and possible causes related to the respective dosage form [17]. However, the EsoCap system represents a completely novel dosage form. It is essential that a special application cup is used. After intake, the retainer, which was necessary to activate unrolling of the film, remains in the throat for a moment. This could lead to a short-term foreign body sensation with nausea in the subject. Questionnaires with different focuses have been established to evaluate the acceptability of oral dosage forms [12,15,18]. To verify the basic functionality of the EsoCap system principle, it is necessary to verify the mucoadhesive polymer film’s presence in the esophagus. For this reason, the study was aimed at showing the novel EsoCap system’s in vivo functionality in two different setups, in 12 healthy volunteers, using Magnetic Resonance Imaging (MRI) as a non-invasive technique. An MRI contrast-enhanced polymer film that could be visualized in MRI was used to achieve this aim. Two study setups were performed. The dosage forms in the study setups differed in terms of their density to investigate its influence on acceptance and functionality. The system’s swallowability and acceptance were determined by means of a standardized questionnaire. The questionnaires and MRI scans were statistically analyzed for differences between the study setups.

## 2. Materials and Methods

### 2.1. Materials

All materials used were supplied in pharmaceutical or food-grade quality. The hard gelatin capsules (size 00; length 23.3 mm; diameter 8.2 mm) were purchased from Wepa (Hillscheid, Germany). Polyvinyl alcohol (PVA 18-88) was provided by Merck (Darmstadt, Germany). The glycerol needed as a plasticizer for film production was obtained from Caelo (Hilden, Germany). Demineralized water was used as a solvent. Food-grade polylactic acid filament from Formfutura (Nijmegen, the Netherlands) was being used for the production of the drinking cup and applicators. 3D printing of the drinking cup and applicators has been described previously [8]. Hibiscus tea was purchased from Spinnrad (Bad Segeberg, Germany) as a contrasting agent for MRI. A food-grade polyester string used (hereinafter referred to as “retainer”) was purchased from Westmark (Lennestadt-Elspe, Germany). Calcium dihydrogen phosphate and croscarmellose sodium were supplied by JRS Pharma (Rosenberg, Germany), magnesium stearate by Sigma Aldrich Chemie (Taufkirchen, Germany) and iron oxide by Caelo.

### 2.2. MRI Contrasting Film

A concentrate of hibiscus tea was prepared and used as a solvent for the manufacture of MRI-contrasting mucoadhesive films. Tea prepared from *Hibiscus sabdariffa* L. hibiscus calyces is described in the literature as a MRI negative contrast agent for oral use [19]. The films, made of PVA (18%), glycerol (2%), aqueous hibiscus concentrate (75%) and ground hibiscus tea (5%), were prepared using solvent casting technology. Ground hibiscus tea (50 g) was extracted using 200 mL of hot water for 12 h and then centrifuged (3000 rcf; 60 min, mod. 5702 R centrifuge, Eppendorf, Hamburg, Germany). The extract was mixed with the other components in a laboratory glass bottle and mixed by means of a magnetic stirrer at 90 °C, in a water bath, with constant stirring for 6 h. The mixture was then cold stirred at 50 rpm and the film laminates were prepared on a liner a maximum of 24 h before in vivo testing. A layer height of 1000 μm, at a speed of 10 mm/s, by means of a motorized film-spreading device (296–CX4E, Eckla, Bretzfeld, Germany) was used. The drying of the laminates was carried out at room temperature (relative humidity approx. 30%). Narrow 0.4 cm × 22.0 cm strips were cut from the film laminates (mass 175 mg) and stored in airtight aluminum composite bags until further use.

### 2.3. Preparation of the Sinker

A powder mixture of calcium dihydrogen phosphate (93.6%), sodium croscarmellose (5.0%), magnesium stearate (1.0%), iron oxide (0.4%) was processed into the used sinker. A homogeneous powder mixture was obtained by sieving the individual components in a sandwich process, with a subsequent mixing process at 49 rpm for 5 min, using a TURBULA^®^ mixer (Willy A. Bachofen AG, Muttenz, Switzerland). A single punch tablet press (KP2, VEB Kombinat NAGEMA, Dresden, former German Democratic Republic) was used to compress the sinker (diameter: 7.00 mm, 8.50 mm, weight: 515 mg).

### 2.4. Design and Preparation of the EsoCap System

The EsoCap system tested generally consists of a slit hard gelatin capsule (size 00), with a rolled-up film in this capsule. The density of this system was 0.4 g/cm^3^ (study setup A). The retainer, which is needed to trigger the unwinding mechanism, was fixed to the end of the film coming out of the capsule by means of a liquid polymer mass. The free end of the retainer was connected to a special 3D-printed applicator. This applicator was connected to a 3D-printed drinking cup filled with water, just before the EsoCap system was applied. All 3D-printed components were produced using a fused deposition modelling 3D printer (Ultimaker 3, Ultimaker BV, Utrecht, the Netherlands). To increase the system’s density to about 1 g/cm^3^, a compressed tablet (about 500 mg), hereinafter referred to as the “sinker”, was introduced into the capsule (study setup B). Figure 1 shows pictures of the different dosage forms used in both study setups.

### 2.5. Ethics

The in vivo study was performed according to the Declaration of Helsinki, Good Clinical Practice and the German MPG §23b. All documents related to the study, including questionnaires, were accepted by the Ethics Committee at University Medicine Greifswald (Germany) prior to conduct of the study (BB 170/18b). All subjects provided written informed consent for study procedures, including MRI, and were insured against any harm caused by study procedures and commuting accidents. Subjects received an appropriate expense allowance for participation.

### 2.6. Subjects

Twelve healthy human subjects participated in the study. The study collective was half male and half female, who had a mean age of 24.5 ± 3.1 years and a mean BMI of 23.0 ± 2.3 kg/m^2^. To ensure good health, subjects were screened based on their medical history and physical examinations. Exclusion criteria for MRI imaging for example were metallic implants. FDA (Guidance for Industry: Food-Effect Bioavailability and Fed Bioequivalence Studies) and EMA (Guideline on the Investigation of Bioequivalence) guidelines for bioavailability and bioequivalence studies set the framework for inclusion criteria. No subjects were with gastrointestinal disorders, previous gastrointestinal surgery, or a history of alcohol or drug abuse were included. Subjects abstained from alcohol for 48 h before study procedures and took no medication known to affect GI physiology. Female volunteers had to perform a urine pregnancy test to exclude gravidity. No other food or drink but the water provided for intake of the EsoCap system were allowed during the study procedures.

### 2.7. Study Protocol

As already mentioned, the EsoCap system device was tested in two different setups. On study days 1–3 (setup A) the system consisted only of the rolled polymer film inside the capsule. In contrast to that, the capsule additionally included a sinker on study days 4–6 (setup B). Thus, each setup was tested in three replicates in all subjects, with no specific washout period. The study was performed at the Institute of Diagnostic Radiology and Neuroradiology (University Medicine Greifswald, Greifswald, Germany).

Before administration of the EsoCap system using the specific applicator, reference images of the esophageal region were taken to ensure that later contrast enhancement was related to the polymer film and not to other food residues, surrounding tissues, or artefacts. The subjects were instructed in detail before taking the EsoCap system. 

As previously described, the EsoCap system applicator was placed on a specialized cup before administration. The cup contained 100 mL water for intake of the capsule in an upright position. Administration was performed directly in front of the MRI scanner immediately after the reference images had been taken. As described by Krause et al. and mentioned above, the polymer film was attached to a retainer that itself was attached to the applicator [8]. By swallowing the capsule, together with the flush of water from the cup, the retainer tightened and pulled out the polymer film from the capsule. The film unrolled and adhered to the esophageal mucosa due to its mucoadhesiveness. To prevent sticking, swelling or dissolving of the components, which could have blocked the system’s function, it was important for the EsoCap system to be swallowed quickly and in a fluid movement. After successful completion of the swallowing process as subjectively perceived by the subject, the applicator was detached from the cup. The retainer is still attached to the mouthpiece hanging through the throat to the—ideally—unrolled film in the esophagus. 

Subjects were positioned in the MRI after ingestion and were required to wait at least 3 min for the bond between the retainer and polymeric film to swell and dissolve. The retainer was pulled out from the gel formed after the first taken recording by the subjects Images were taken at 2 min, 5 min, 10 min and 15 min after intake, with the subjects remaining in the supine position in the MRI for the entire period. Several time points for recordings were chosen to match the moment of best contrast, of the partially swelling/moisture-dependent hibiscus tea film. At the end of the imaging process, the subjects were given a standardized questionnaire to evaluate acceptability by means of swallowability and negative sensations during intake (the questionnaire is shown in supplementary material). Water was provided ad libitum after completion of the measurements. 

Subjects were able to rate the amount of co-administered water by a visual analogue scale, with the best result in the middle, with a 50% score representing an amount of fluid that was “just right” for intake of the EsoCap system. Higher scores would represent excess volume administered and, accordingly, lower scores insufficient volume.

The foreign body sensation during and after intake was to be evaluated by the subjects. Questions were also asked about possible pain and choking impulses that could potentially have been caused by the retainer, for example. In addition, the swallowability was evaluated in general an analog scale was used for scoring as a semi-quantitative tool for subjective evaluation of the strength of sensations such as pain. The best result was 0 (e.g., no choke impulse or swallowability like water), and the worst was 100 (e.g., vomiting or impossibility to swallow). The analog scale was also used to determine swallowability, as this kind of questionnaire is a common approach to evaluate swallowability [20,21,22,23].

### 2.8. Magnetic Resonance Imaging

A Siemens MAGNETOM Aera tomograph (Siemens Healthineers, Erlangen, Germany), with a magnetic field strength of 1.5 Tesla, was used for acquisition of magnetic resonance images of the esophageal area.

Subjects were placed in supine position and a six-element phase array abdominal receiver coil, four spine coils inside the MRI desk, and a head/neck cage coil were used for signal detection. Coil selection was automatically performed using acquisition software Syngo MR E11 (Siemens), depending on placement of field of view (FOV). Strongly T1 weighted sagittal and transversal images were acquired for visualization of the contrast-enhanced polymer film in the esophagus. Imaging was performed using a VIBE sequence, with a repetition time of 3 ms, an echo time of 1.4 ms, a slice thickness of 2.5 mm, no interslice gap and a flip angle of 30°. A slice oversampling of 30–50% was performed to avoid folding artefacts. The number of slices, phase oversampling and thus acquisition time were adapted according to individual anatomy. All acquisitions were performed within a single breath hold to reduce motion artefacts.

### 2.9. Image Analysis

The images were evaluated using Horos v2.2.0 freeware (The Horos Project). The first aim was to detect the unrolled polymer film in the esophagus, as well as the susceptibility artefact caused by the iron oxide included in the sinker in setup B. The unrolled length of polymer film was measured manually by three independent trained observers. The mean was calculated from these three measurements, as representative length, if differences between observations were less than 10% of measured length. Results were under discussion until consensus was reached when major differences occurred.

The last imaging time point (15 min) was used to evaluate the duration of visibility of the film at the application site. To calculate the percentage of film unrolled, the visible film length was divided by the total length (22 cm) of the EsoCap film used. 3D reconstructions and signal-intensity projections were used for visualization.

### 2.10. Statistics

To evaluate the significance of differences between unrolled film length between setup A without sinker and setup B with sinker data were tested for normal distribution by Shapiro-Wilk test and Kolmorov-Smirnov test. Since data were not of gaussian distribution, data were log transformed with equation ln(x+1), to adapt for values of x = 0. Log-transformed data conformed normal distribution criteria and were tested by paired t-test subsequently with *p* < 0.05 assuming a statistical significant difference between both groups. To evaluate the significance of differences between questionnaire scores on swallowability patterns between setup A without sinker and setup B with sinker data were also treated the same way as they also had no gaussian distribution, but data also had no log-normal distribution so that log transformation was not sufficient. Due to pronounced positive skewness of some data sets irrespective of log-transformation, Wilcoxon signed rank test was not possible. Thus, two-sample paired sign test with *p* < 0 assuming a statistical significant difference between both groups was performed on raw data without previous transformation. Graphical depictions and statistical calculations of data were prepared with OriginPro 8.5.1G (OriginLab Corporation, Northampton, MA, USA).

## 3. Results

The capsules could be successfully swallowed in 71 out of the 72 applications. Due to incorrect assembly of the system in one case in study setup A, the capsule stuck in the applicator and could not, therefore, be swallowed.

**Figure 2:**
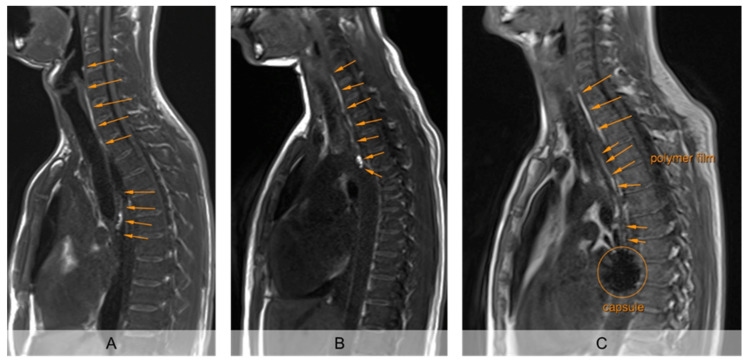
Exemplary representation of sagittal imaging 5 min after application of the EsoCap system. (**A**): (Almost) completely unrolled film (study setup A, without additional sinker). (**B**): Incompletely unrolled film, with particularly intense signal of the unrolled film in the capsule (study setup A, without additional sinker). (**C**): (Nearly) completely unrolled film, using sinker, loaded with iron oxide for signal extinction (study setup B).

The unrolled PVA film in the esophagus was clearly detectable from the first recording 2 min after intake in strongly T1 weighted MRI, so that its unrolled length was measurable (Figure 2). The mucoadhesive PVA film contrasted by hibiscus tea was clearly visible from the surrounding tissue in all subjects on all days. Moreover, the sinker loaded with iron oxide used in setup B showed a clear signal extinction on MRI, so that the fate of the carrier capsule could also be evaluated (Figure 2C).

**Figure 3:**
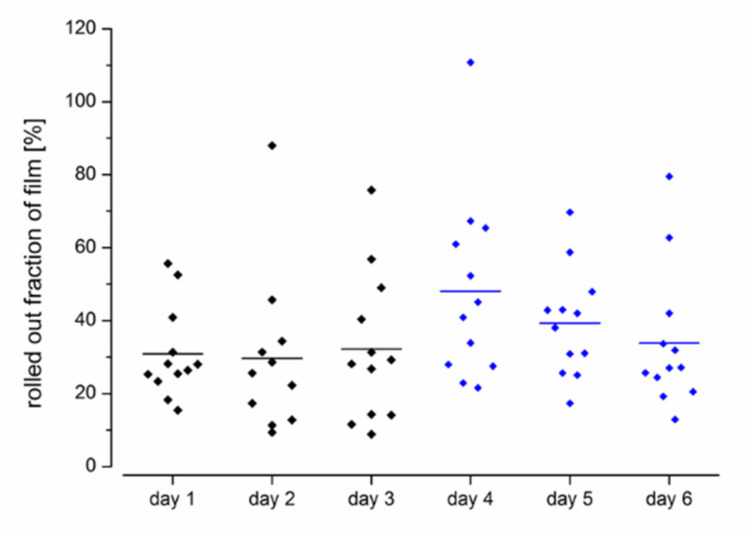
Unrolled film length from the EsoCap system after administration by healthy volunteers (*n* = 12) (study setup A: day 1–3; study setup B: day 4–6).

Evaluation of the MRI images showed that the average film length unrolled from the EsoCap system on the individual days in setup A without sinker was between 6.5 and 7.1 cm (Figure 3). Thus, approximately 15 cm remained in the capsule, which was visible as a particularly bright spot on the MRI images (Figure 2A,B). In study setup B, on average 9.6 cm, 7.9 cm and 6.8 cm were unrolled, with highest values on day 4. In general, the use of a sinker significantly increased the unrolled length from 6.8 ± 3.9 cm (*n* = 36) in all administrations in setup A to 8.1 ± 4.1 cm (*n* = 36) in setup B (paired *t*-test, *p* < 0.05). The iron oxide-loaded sinker made it particularly easy to localize the capsule in the subject and to evaluate the unrolled film length.

**Figure 4:**
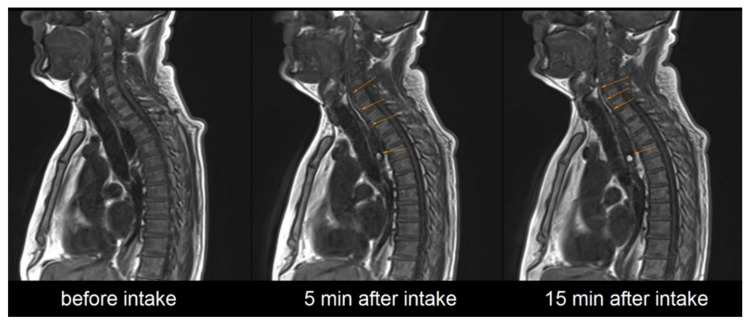
Exemplary representation of sagittal image acquisition before, 5 min after and 15 min after intake of the EsoCap system.

It needs to be highlighted that after successful application the contrasting film could be detected on MRI in all cases. In 25 out of 36 administrations the contrast-enhanced polymer film was still detectable in the esophagus, even 15 min after administration of the EsoCap system, as shown in Figure 4.

**Figure 5:**
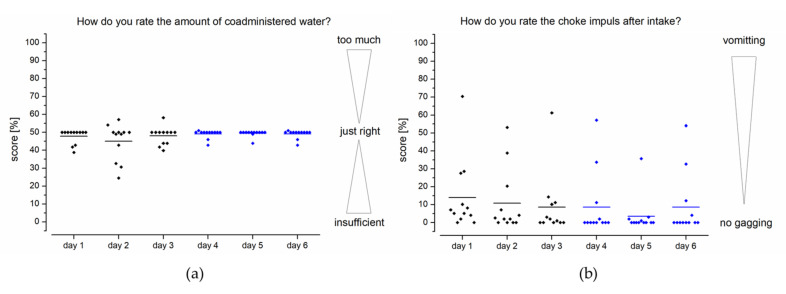
Questionnaire feedback by healthy volunteers (*n* = 12) on the volume of water offered (**a**) and choke impulse after application of the device (**b**). Study setup A: days 1–3; study setup B: days 4–6.

Feedback from assessment of the volume of water available (100 mL) showed that in the first three days (study setup A), many subjects rated the amount of fluid as “just right” (Figure 5a). However, some subjects would like to have a little more fluid to take the novel device. Especially on day 2, there was an increased scattering of feedback. With the use of an additional sinker (setup B/days 4–6), the variability of feedback could be reduced to the positive, so only two test persons would like to have a greater volume of liquid.

The feedback on the choke impulse feeling after application was similarly positive, as for assessment of the amount of fluid available. Most subjects described no or little choke impulse after film application using the EsoCap system (Figure 5). In setup A, a greater scattering of feedback can be clearly seen, especially on day 1. Nonetheless, nine out of twelve subjects indicated that their choke impulse was about or below 10% of the rating scale. During further study days, in general the number and severity of choke impulses continued to decrease. The choke impulse was further reduced with the higher density capsules and was only occasionally reported by the subjects. Across all days it was observed that only three of the twelve subjects experienced a distinct choking sensation. In the case of the increased density capsules, most of the subjects even reported no choking at all (score 0). Vomiting did not occur in any case during the study, which is consistent with the few reports of choking.

**Figure 6:**
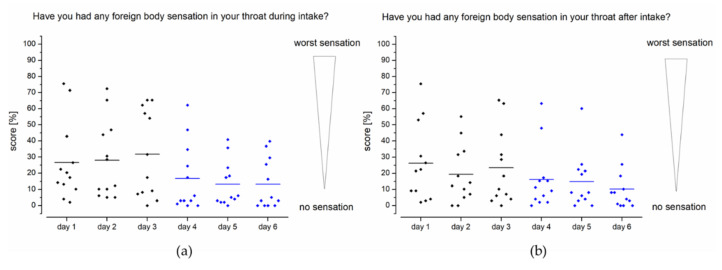
Questionnaire feedback from healthy volunteers (*n* = 12) on foreign body sensation during intake (**a**) and after intake (**b**). Study setup A: days 1–3; study setup B: days 4–6.

In addition to general evaluation of choke impulses after intake (Figure 5b), the foreign body sensation during (Figure 6a) and after taking (Figure 6b) the device was determined. In study setup A, the mean score for foreign body sensation was 29%. A foreign body sensation with a score below 10% was reported during intake of the EsoCap system for approximately half of the applications. In study setup B, scores for foreign body sensations were significantly less pronounced (two-sample paired sign test, *p* < 0.0001).

The scores obtained for the difference between the study setups for foreign body sensation after intake of the capsule are quite comparable to foreign body sensation during intake. Again, the capsule with the higher density (study setup B) received much better scores than the low-density capsules (study setup A). While assessment of the device in setup A was largely evenly distributed over the rating scale, it can be seen in setup B that use of a sinker significantly reduced the foreign body sensation in almost all subjects (two-sample paired sign test, *p* < 0.05). For example, on day 4, 10 out of 12 subjects rated the foreign body sensation as less than the general mean on that day (16%), and only two subjects had a greater foreign body sensation.

**Figure 7:**
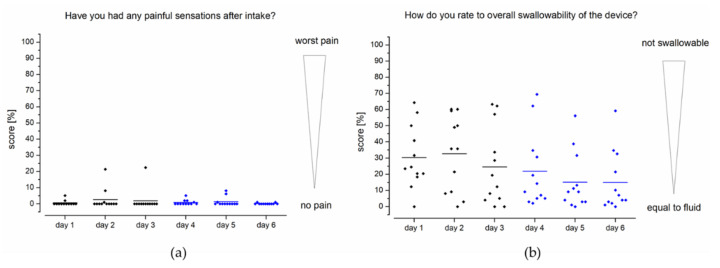
Feedback from healthy volunteers (*n* = 12) on painful sensations after intake (**a**) and the device’s overall swallowability (**b**). Study setup A: days 1–3; study setup B: days 4–6.

As can be seen in Figure 7a, no relevant pain events were reported in connection with intake of the EsoCap system. Of particular interest is the assessment of one subject each on days 2 and 3, who reported a pain sensation with a score of about 22% after ingestion on these two days. In study setup B using the sinker-modified EsoCap system, isolated reports of minimal painful events of less than 8% were reported by individual subjects. Overall, the scoring for pain was at an extremely low level of less than 1%. The score for overall swallowability of the EsoCap system (Figure 7b) was 29% on the rating scale in setup A and 17% in setup B. The use of a sinker to adjust the EsoCap system’s density led to a significant increase in swallowability between study setups A and B (two-sample paired sign test, *p* < 0.0001). 

## 4. Discussion

The application of films for local drug therapy in the esophagus could represent a great opportunity to improve the treatment options for affected patients of different diseases. The esophagus is a particularly difficult site of application to treat with locally effective drugs because of the ultra-short transit times [1,2]. The novel EsoCap drug delivery system technique is designed to enable a highly variable platform for local therapy targeted to the esophagus [8]. In the reported study, we tested acceptance of the novel application system in 12 healthy volunteers, with 6 applications each in two different study setups. The films were successfully placed in 71 of 72 administrations (one application failed in study setup A due to the capsule sticking to the applicator), proving the principal functionality. In addition, it was possible to visualize the mucoadhesive film using a hibiscus tea concentrate, combined with strongly T1-weighted VIBE sequences. Clear visualization of the film in the MRI was a major challenge due to the voxel size (resolution of the MRI used). The demands on the film’s visibility in MRI and the thin nature of a film represent a general challenge that was successfully solved using hibiscus teas. This shows that hibiscus tea is an exciting candidate for labelling of dosage forms in future studies, due to its general applicability and safety as a food substance.

Many subjects reported that they felt no, or almost no problems during application of a film using the EsoCap system. However, there were a few subjects who described application of the film as a challenge, as is described for other dosage forms, such as by Schiele et al. [17]. Young women especially often have major problems in taking large oral dosage forms [17]. The subjects in this study were a young collective of 24.5 ± 3.1 years and consisted of half females and half males. Thus a healthy, but nevertheless particularly critical, subject collective participated in this study to test this novel drug delivery concept. Diseases could have a further influence on swallowability. In addition, the carrier capsule of size 00 used is a decidedly large-volume dosage form. Hansen et al. demonstrated the ability of young subjects to learn to take oral dosage forms [16]. Acceptance could be further increased if the subjects were trained beforehand, by taking placebo tablets or capsules with an identical density, for example, using the EsoCap system cup [16,17,24,25].

The use of an additional sinker in study setup B significantly improved overall swallowability, as well as the foreign body sensation during application (two-sample paired sign test, *p* < 0.05), compared to study setup A. The significantly more comfortable mucoadhesive film application is most likely due to the overall system’s increased density. In study setup A, the density of the total system (capsule and film) was about 0.4 g/cm^3^. Thus the capsule floated in the oral cavity. The capsule’s buoyancy and head and neck placement due to applicator position can result in reduced swallowability. This problem has already been described in 1968, in a U.S. patent on the “Method of swallowing a Pill” [14]. The neck position is only advantageous for dosage forms with the same or a higher density compared to water. By increasing system density to 1.0 g/cm^3^ by means of a sinker (study setup B) and thus improving swallowability, overall swallowability could be significantly improved and foreign body sensation in the test subjects during and after application significantly reduced.

Another way to improve swallowability could be to reduce the size of the EsoCap system [17]. Using a smaller capsule, for example capsule size 0, while maintaining the same or higher density, could further increase acceptability. In this study it was not possible to use a smaller capsule size due to the particularly thick film (220 μm) required for visibility in MRI. A film intended for therapeutic use could have approximately half the thickness, so a reduction in capsule size is easily possible. Moreover, use of thinner films could increase the length of the rolled film possible. In the present study, the length was only 22 cm, which would not be enough to cover the whole esophagus, which typically has a length of about 25 cm. These few centimeters could easily be included by the use of thinner films. In addition, the study used a water-insoluble polyester retainer that had to remain in the oral cavity for more than 3 min after application of the film, until sufficient swelling or erosion of the retainer-film connection had occurred. This insoluble retainer potentially led to increased foreign body sensation. The use of a thin and highly flexible retainer, which is rapidly water-soluble after application, could further increase acceptance.

In both study setups, the unrolled length of the particularly thick and less flexible contrasting films averaged 7.5 cm and was not, therefore, completely unrolled. In addition, there is no clear trend in the unrolled film length over the days. Nonetheless, we were able to show that inclusion of a sinker significantly increased the mean unrolled fraction from 31 ± 18% in setup A, to 40 ± 21% in setup B with the sinker (paired *t*-test, *p* < 0.05). The high variability in the unrolled fraction might be related to the manual production of very thick films, manual slitting of the capsules and manual assembly of the more complex system. The use of thinner films, uniformly slotted capsules, and automation of the manufacturing process could result in the film’s significantly lower unwinding resistance from the capsule. The film’s unwinding length could be increased as a result of these developments. From a therapeutic point of view, it might not be a disadvantage that the EsoCap system gets stuck in the esophagus and the film is not completely unrolled and placed in the esophagus [26]. If the system is not completely unrolled, for example because the capsule gets stuck in the esophagus, the unrolled setup of the film forms a kind of deposit. The capsule and the sinker disintegrate within a short time. The film polymer is slowly dissolved by the mucosal moisture, so that it changes to a gel-like consistency. This highly viscous and gel-like deposit is transported distally towards the stomach due to peristaltic movements in the esophagus and could thus provide long-lasting local drug therapy throughout the esophagus. The sinker should disintegrate within a short time due to the quantities of disintegrants and should not cause any irritation. The highly promising EsoCap system’s clinical benefit needs to be determined in further studies with diseased subjects.

## 5. Conclusions

In this study, 71 out of 72 films were successfully placed locally in the esophagus of 12 healthy volunteers using the novel EsoCap system. The dosage form’s functionality and acceptance could be confirmed by MRI and standardized questionnaires. In addition, it was possible to demonstrate increased acceptance of the EsoCap system, modified by an additional weight. Due to the multitude of possible applications, the EsoCap system represents an exciting, forward-looking and highly variable platform for the local application of various films in the esophagus. A further increase in acceptance of the technology can be expected through possible modifications to the system and training of test persons. Further in vivo studies with drug-loaded films will be necessary to demonstrate this promising EsoCap system’s clinical benefit.

## Data Availability

The MRI data that support the findings of this study may be available on request from the corresponding author W.W., depending on requested information. The data are not publicly available due to them containing information that could compromise research participant privacy or consent according to German Data Protection Act. Explicit consent to deposit raw-sequencing data was not obtained from the subjects. Data from in vitro characterization and results from MRI study are available on request from the corresponding author W.W.

## Supplementary Materials

The following are available online at https://www.mdpi.com/article/10.3390/pharmaceutics13060828/s1.
