# Supplementary material for: Functionality and Acceptance of the EsoCap System—A Novel Film-Based Drug Delivery Technology: Results of an In Vivo Study
Source: Pharmaceutics. 2021 Jun 2;13(6):828. doi: 10.3390/pharmaceutics13060828 (PMC8227674; doi:10.3390/pharmaceutics13060828)
Supplement: Supplementary file 1 [file pharmaceutics-13-00828-s001.zip › Questionnaire.pdf]

Fragebogen

In vivo Evaluierung eines neuen Systems zur Platzierung von Polymerfilmen im Ösophagus

---

Datum:

Versuchsnummer:

Proband:

Wie gut ließ sich die Arzneiform schlucken?

Kein Unterschied zu reiner Flüssigkeit — Gar nicht schluckbar

Wie ausreichend empfanden sie die Wassermenge bei Einnahme?

zu wenig — genau richtig — zu viel

Hatten Sie während der Applikation ein Fremdkörpergefühl im Rachenhalsbereich?

Kein Fremdkörpergefühl — Am stärksten vorstellbares Fremdkörpergefühl

Hatten Sie nach der Applikation ein Fremdkörpergefühl im Rachenhalsbereich?

Kein Fremdkörpergefühl — Am stärksten vorstellbares Fremdkörpergefühl

Hatten Sie nach der Einnahme Schmerzen im Rachenhalsbereich?

Keine Schmerzen — Am stärksten vorstellbare Schmerzen

Hatten Sie nach der Einnahme einen Würgereiz?

Kein Würgereiz — Erbrechen

Wurde abschließend Wasser nachgetrunken? ☐ Nein

☐ Ja: ___________ mL

Kommentare:
